# Children and natural disasters

**DOI:** 10.1080/20008198.2018.1500823

**Published:** 2018-08-15

**Authors:** Atle Dyregrov, William Yule, Miranda Olff

**Affiliations:** a Center for Crisis Psychology, Faculty of Psychology, University of Bergen, Bergen, Norway; b Institute of Psychiatry, Psychology and Neurosciences, King’s College, London, UK; c Department of Psychiatry, Academic Medical Center at the University of Amsterdam, Amsterdam, The Netherlands; d Arq Psychotrauma Expert Group, Diemen, The Netherlands

**Keywords:** Natural disasters, resilience, children, adolescents, trauma, PTSD, Desastres naturales, resiliencia, niños, adolescentes, trauma, TEPT

## Abstract

The number of children affected by natural disasters each year is alarmingly high and can be expected to rise as climate change continues. The mental consequences have been documented not only in the rates of post-traumatic stress symptoms and post-traumatic stress disorder, but also for depression and other mental health problems. To contribute towards the knowledge in this area, this special issue of the *European Journal of Psychotraumatology* focuses on how children can be prepared for natural disasters and the mental health aspects of such events. It includes articles on communicating risks to children, involving children in disaster risk reduction, and the mental health consequences for children from an earthquake, a volcanic eruption, a flood and a hurricane. In this special issue specifically focusing on children and natural disasters, we hope to enhance our understanding of some of the complex pathways and pave the way for improving our interventions.

## Abstract (es)

La cantidad de niños afectados cada año por desastres naturales es alarmantemente alta y es esperable que aumente a medida que el cambio climático continúa. Las consecuencias mentales se han documentado no solo en tasas de Síntomas de Estrés postraumático (SEPT) y Trastorno por Estrés postraumático (TEPT), sino en depresión y otros problemas de Salud Mental. Para contribuir a un mayor conocimiento, este ejemplar especial del Journal Europeo de Psicotraumatología se focaliza en como pueden prepararse los niños para los desastres naturales y los aspectos de salud mental de dichos eventos. Incluye artículos sobre la comunicación de riesgos a los niños, de involucrarlos en la reducción de riesgo de desastres, y las consecuencias de salud mental para niños por un terremoto, una erupción volcánica, una inundación y un huracán. En este ejemplar especial focalizado específicamente sobre niños y desastres naturales esperamos haber mejorado nuestra comprensión de algunas de los complejos senderos y pavimentado el camino para mejorar nuestras intervenciones.

## 儿童与自然灾害

每年受自然灾害影响的儿童数量惊人，并预计会随着气候的持续变化增加。有记录表明，由此带来的心理后果不仅仅是创伤后应激症状（PTSS）和PTSD的发生率，也包括抑郁症和其他心理健康问题。为了增加对其了解，本期《欧洲心理创伤学杂志》特刊重点关注儿童如何为自然灾害做好准备，和此类事件对心理健康影响。特刊文章主题包括有：与儿童沟通的风险，让儿童参与降低灾害风险的措施，以及地震、火山爆发、洪水和飓风等对儿童的心理健康影响。在这个特别关注儿童和自然灾害的特刊中，我们希望增加对一些复杂路径的理解，并为改进干预措施铺平道路。

It is estimated that about 175 million children per year will be affected by natural disasters attributed to climate change (Codreanu, Celenza, & Jacobs, 2014). Globally, children are estimated to bear 88% of disease due to climate change, and the poorer they are the greater their burden (Philipsborn & Chan, 2018). The number of children seriously injured or dying from such disasters each year is unknown but immense, and added to this are the mental consequences. Estimates are that 5–43% of affected children will experience post-traumatic stress disorder (PTSD), and many suffer from depression, anxiety or other mental health disturbances (Kar, 2009). With climate change, the mental health burden on children and families can be expected to rise. Drought and poverty may lead to an exodus of people from many areas, with social tensions rising as masses of people move to seek a better future.

Children who experience drought, hurricanes, earthquakes, landslides, bush fires and other natural disasters need parental care and societal support to mitigate the stressors they encounter. Can they be prepared for such events? Can better systems be developed to meet their needs? Can children themselves take an active part in preparation and rebuilding hope after disastrous events? Is social support available and will children make use of it? The questions are many and the knowledge is sparse.

Trauma and disasters occur around the world (Schnyder, 2013) and need to be met with a public health approach (Magruder, Kassam-Adams, Thoresen, & Olff, 2016). The *European Journal of Psychotraumatology* has published extensively on global mental health issues with several special issues: Trauma occurs in social contexts (Sijbrandij & Olff, 2016); Global mental health and trauma (Purgato & Olff, 2015); Trauma and adversity among populations in transition (Hall & Olff, 2016); and Traumatized refugees: identifying needs and facing challenges for mental health care (Knaevelsrud, Stammel, & Olff, 2017). Research on traumatized children is increasing (e.g. Dyb & Olff, 2014; Kristensen, Dyregrov, & Dyregrov, 2018; Strøm, Schultz, Wentzel-Larsen, & Dyb, 2016; Yule, Dyregrov, Raundalen, & Smith, 2013); however, still little is known about how children cope with natural disasters. In this special issue we focus on children and natural disasters. It includes six articles spanning different themes and different continents, from an earthquake in China, through a hurricane and a flood in the USA and India, to a volcanic eruption in Iceland.

Cheng, Liang, Fu, and Liu (2018) examined the relationship between PTSD and depressive symptoms following the earthquake in China in 2008 that killed almost 70,000 people and injured close to 375,000, with around 18,000 recorded as missing. After studying 300 children assessed at four follow-up points over more than 4 years, they concluded that the causal relationship between PTSD and depressive symptoms changed over time. The effect of PTSD tended to decrease over time, while the opposite was true of depression. Regarding risk, they found that females with poor parental relationships and high trauma exposure were more in need of intervention.

Hove Midtbust, Dyregrov, and Wittrup Djup (2018) focus on how best to prepare children and young people to respond to major risks. In theory, they could be made more aware of the risks and how best to react to them. In practice, research indicates that knowledge of preparedness is not strongly related to undertaking appropriate behaviour. While schools are in a good position to teach about risk awareness and safety behaviours, parents also need to be fully involved to maximize the chance that young people have better chances of survival in the face of disasters.

Krishna, Ronan, and Alisic (2018) report on the effects of the Chennai flood of 2015 on children’s adjustment as related by adults. The qualitative study found that the community was unprepared for the effects of the flooding, and post-disaster behaviour was greatly affected by the Indian caste system and the enduring poverty. However, there were encouraging indications that children did undertake important rescue behaviour and would do more with better preparation.

Pfefferbaum, Pfefferbaum, and van Horn (2018) develop this theme of children being active agents in their own lives. A review of various theoretical models indicates that children can be involved in disaster risk reduction. This should improve their interpersonal skills as well as help them to develop particular skills in anticipating and responding to various disasters. The challenge at present is to document and evaluate ways of engaging children in such behaviour.

Lai, Osborne, Piscitello, Self-Brown, and Kelley (2018) studied the relationship between social support and post-traumatic stress symptoms (PTSS) among children exposed to Hurricane Katrina in 2005. They tested social causation and social selection models simultaneously in a longitudinal study and found support for the social selection model. This means that those with more PTSS either avoid sources of social support, or perceive less support as available, or are selected out of supportive relationships. Finding that PTSS generally precedes decreases in social support, this study points to prioritizing the reduction of PTSS to prevent cascading effects on social support.

What is the long-term health impact of natural disasters? Hlodversdottir and colleagues (2018) studied adults in Iceland following the dramatic eruption of Eyjafjallajökull in 2010. They asked the adults – both those exposed to the volcanic eruption and those not exposed – about the psychological and physical effects on their children, both in 2010 and in 2013. Exposed children were reported to have increased respiratory symptoms and anxiety. Those whose homes were damaged evidenced increased distress. The level of distress had not fallen by 3 years later. As in other studies reported in this special issue, it is unfortunate that children were not directly interviewed about their own reactions.

This special issue is just a start, trying to bring together research that focuses on this understudied area. We may increase our knowledge by mutual learning and collaboration around the world (Schnyder et al., 2017) and by acknowledging cultural sensitivity (Schnyder et al., 2016). Disasters will usually not result in most children developing mental health difficulties in need of clinical intervention. However, even a low percentage of PTSD, depression or other clinical disorders may involve a large number of children following disasters. If a million children are heavily impacted by an earthquake and 10% of them develop PTSD, we have 100,000 children who may benefit from mental health interventions. Future studies should look at screening methods that can be used to identify children with clinical needs (e.g. Verlinden et al., 2015) and include studies on the use of groups that can reach many children, and how and where best to run them. Studies should look at early preventive interventions that reach out to all affected children and their families to lower the number of children needing specific therapeutic interventions (e.g. Kassam-Adams, 2014). Research that focuses on identifying and evaluating cost-effective interventions that can easily be scaled up to meet large demands following disasters is needed. Just before the Asian tsunami of 2004, Mollica and co-workers (2004) argued that every country should prepare to respond to natural disasters with screening tools, training of staff and plans for research. Only by forward planning and ready funding will we ever be able to reduce the impact of disasters on the mental health of our children.
